# Intracellular diffusion restrictions in isolated cardiomyocytes from rainbow trout

**DOI:** 10.1186/1471-2121-10-90

**Published:** 2009-12-17

**Authors:** Niina Sokolova, Marko Vendelin, Rikke Birkedal

**Affiliations:** 1 Laboratory of Systems Biology, Institute of Cybernetics, Tallinn University of Technology, Akadeemia 21, 12618 Tallinn, Estonia

## Abstract

**Background:**

Restriction of intracellular diffusion of adenine nucleotides has been studied intensively on adult rat cardiomyocytes. However, their cause and role *in vivo *is still uncertain. Intracellular membrane structures have been suggested to play a role. We therefore chose to study cardiomyocytes from rainbow trout (*Oncorhynchus mykiss*), which are thinner and have fewer intracellular membrane structures than adult rat cardiomyocytes. Previous studies suggest that trout permeabilized cardiac fibers also have diffusion restrictions. However, results from fibers may be affected by incomplete separation of the cells. This is avoided when studying permeabilized, isolated cardiomyocytes. The aim of this study was to verify the existence of diffusion restrictions in trout cardiomyocytes by comparing ADP-kinetics of mitochondrial respiration in permeabilized fibers, permeabilized cardiomyocytes and isolated mitochondria from rainbow trout heart. Experiments were performed at 10, 15 and 20°C in the absence and presence of creatine.

**Results:**

Trout cardiomyocytes hypercontracted in the solutions used for mammalian cardiomyocytes. We developed a new solution in which they retained their shape and showed stable steady state respiration rates throughout an experiment. The apparent ADP-affinity of permeabilized cardiomyocytes was different from that of fibers. It was higher, independent of temperature and not increased by creatine. However, it was still about ten times lower than in isolated mitochondria.

**Conclusions:**

The differences between fibers and cardiomyocytes suggest that results from trout heart fibers were affected by incomplete separation of the cells. However, the lower ADP-affinity of cardiomyocytes compared to isolated mitochondria indicate that intracellular diffusion restrictions are still present in trout cardiomyocytes despite their lower density of intracellular membrane structures. The lack of a creatine effect indicates that trout heart lacks mitochondrial creatine kinase tightly coupled to respiration. This argues against diffusion restriction by the outer mitochondrial membrane. These results from rainbow trout cardiomyocytes resemble those from other low-performance hearts such as neonatal rat and rabbit hearts. Thus, it seems that metabolic regulation is related to cardiac performance, and it is likely that rainbow trout can be used as a model animal for further studies of the localization and role of diffusion restrictions in low-performance hearts.

## Background

In permeabilized preparations of mammalian oxidative muscles such as red skeletal muscle and the heart, diffusion of ADP and phosphate from the surrounding medium to the adenine nucleotide translocase (ANT) in the inner mitochondrial membrane is restricted [1,2]. Especially the diffusion restriction of ADP in adult rat cardiomyocytes has received much attention, because the compromised energetic balance induced by ischemia-reperfusion damage is associated with diminished diffusion restrictions [3,4].

The existence of diffusion restrictions is indicated by that the apparent ADP-affinity of mitochondria *in situ *in permeabilized fibers and cardiomyocytes is much lower (K_M ADP_~300 μM in rat fibers and cardiomyocytes) than the ADP-affinity of isolated mitochondria (K_M ADP _< 20 μM) [5-7]. It was first proposed that this was due to low permeability of the outer mitochondrial membrane, which is partially overcome by creatine kinase (CK) [8,9]. In addition to the outer mitochondrial membrane, some diffusion restrictions also reside in the cytosol. They lead to a preferential metabolic channeling of ADP from ATPases to mitochondria [10]. Likewise, ATP from mitochondria is channeled to the sarcoplasmic reticulum (SR) Ca^2+^-ATPase (SERCA) and myosin ATPase [11]. This led to the hypothesis that cytosolic diffusion restrictions divide cardiomyocytes into subcellular compartments termed "intracellular energetic units" (ICEUs) [12-14]. Their confinement of ADP and ATP in small compartments with short diffusion distances enhances the functional coupling between adjacent energy producing mitochondria and energy consuming ATPases.

It is still not clear what causes the diffusion restrictions in rat cardiomyocytes. The importance of the outer mitochondrial membrane has recently regained attention in studies on isolated brain mitochondria suggesting that its permeability is regulated by tubulin [15,16]. Possible candidates for cytosolic diffusion restrictions are membrane structures such as SR and t-tubules [12,14]. But also cytoskeletal components such as tubulin have been proposed [6]. These structures are rather densely packed in adult rat cardiomyocytes. In contrast, cardiomyocytes from rainbow trout (*Oncorhynchus mykiss*) have a relatively simple morphology. They are only 2-5 μm thick [17,18], have no t-tubules and a sparsely developed SR [19]. Below the sarcolemma is a single ring of myofilaments surrounding a central core of mitochondria [18,20]. Thus, if t-tubules and SR cause diffusion restriction, we would expect diffusion restrictions to be much smaller in trout cardiomyocytes.

Previous studies on rainbow trout permeabilized cardiac fibers suggest that despite their simple morphology, they also have intracellular diffusion restrictions. As in rat permeabilized fibers, their apparent ADP-affinity is much lower than that expected for isolated mitochondria, but it is increased by the addition of creatine. Furthermore, a competitive assay with excess pyruvate kinase activity only partially inhibits mitochondrial respiration [21,22]. However, the results from permeabilized fibers may be affected by incomplete cell separation, and/or clustering of fibers on top of the stirrer during oxygraphy measurements. These are avoided with permeabilized cardiomyocytes, since the single cells disperse evenly in the oxygraphy medium. Rat permeabilized fibers and cardiomyocytes have similar ADP-kinetics [6], which reaffirm the reliability of the results. However, trout permeabilized fibers have given contradictory results, because creatine lowers the apparent K_M ADP _[21,23], but there does not seem to be a mitochondrial CK tightly coupled to respiration [22]. The aim of this study was to control the existence of diffusion restrictions in trout cardiomyocytes by comparing the apparent ADP-affinity in permeabilized cardiac fibers, permeabilized isolated cardiomyocytes and isolated mitochondria. An effect of temperature on diffusion restrictions in terms of apparent K_M ADP _was found previously on both trout and rat cardiac fibers [23,24]. This is important for the rainbow trout which lives at a temperature range of 2-23°C. Furthermore, due to the different temperature sensitivity of metabolism and diffusion speed, the effect of temperature allows us to draw conclusions about the importance of diffusion distance as one of the restricting factors. Therefore, the experiments were performed at 10, 15 and 20°C. Additionally, because CK may play an important role in facilitating ADP-diffusion across diffusion restriction barriers, the experiments were performed in both the absence and presence of creatine.

The experiments were complicated by hypercontraction of isolated trout cardiomyocytes in the R(respiration)-solution used previously for permeabilized fibers. This solution was originally developed for mammalian tissue and will be referred to as "mammalian R-solution". Therefore, we first had to develop a new "fish R-solution" for trout cardiomyocytes, in which they maintained shape and showed stable steady state respiration rates throughout each experiment. The results on cardiomyocytes were different from those on fibers suggesting that results from trout fibers were affected by incomplete separation of the cells. However, the apparent ADP-affinity of cardiomyocytes was still about an order of magnitude higher than that of isolated mitochondria. This indicates that trout cardiomyocytes still have diffusion restrictions despite their low density of intracellular membrane structures. However, in cardiomyocytes, creatine did not lower the apparent K_M ADP _suggesting that there is no mitochondrial creatine kinase tightly coupled to respiration to overcome diffusion restrictions.

## Methods

### Animals

Rainbow trout were obtained from local fish farms (Simuna Ivax OÜ, Lääne-Virumaa, and Forkala OÜ, Roosna-Alliku, Estonia). They were kept in a 1200 liter freshwater tank at 15 ± 1°C, and fed regularly with commercial fish food. They were allowed to acclimatize for at least 3 weeks before the experiments. All procedures were approved by the Estonian National Committee for Ethics in Animal Experimentation (Estonian Ministry of Agriculture). The fish was stunned by a single blow to the head followed immediately by a cut of the spine. The heart was excised and immediately transferred to ice-cold solution to minimize ischemia.

### Preparation of permeabilized cardiac fibers

The excised heart was transferred to ice-cold isolation solution (see composition below). The ventricle was isolated and the spongy layer and compact layer were separated. The spongy tissue was cut into six smaller pieces, which were transferred to fresh S(skinning)-solution in a petri-dish on ice. Each piece was carefully dissected using fine tweezers (Dumont 3, Dumostar, World Precision Instruments) to separate the tissue into thin bundles of cells that were interconnected. The fibers were permeabilized by incubation for 30 min under continuous mixing in S-solution containing 50 μg/ml saponin at 4°C. After this, they were washed twice for 10 min in S-solution without saponin and stored at 4°C in S-solution until use within less than 3 hours.

### Isolation of cardiomyocytes

The isolation of trout cardiomyocytes has been described previously [18]. The heart was excised with atrium and bulbus arteriosus and transferred to ice-cold isolation solution (see composition below). A cannula was inserted through the bulbus arteriosus into the ventricle and the heart was perfused in retrograde manner with isolation solution for 8-10 min to wash the heart free from blood and to lower extracellular Ca^2+^-concentration. After washing, the heart was perfused with isolation solution containing 0.5 mg/ml trypsin, 0.75 mg/ml collagenase A and 0.75 mg/ml BSA for 18-20 min to digest the extracellular matrix and dissociate the cardiomyocytes. After digestion, the heart was taken off the cannula and cut into smaller pieces. The cardiomyocytes were suspended with a 1 ml pipette, where the tip had been cut off in order to minimize mechanical damage. The cell suspension was filtered through nylon tissue, and healthy cells were isolated by sedimentation. The isolation procedure was carried out at room temperature, and the isolated cells were kept in isolation solution at 4°C until use within at least 3 hours.

### Isolation of mitochondria

Mitochondria were isolated by differential centrifugation of tissue homogenate similar to in Bouzidi et al. [25]. The heart was excised and transferred to buffer A (see composition below). The atrium and bulbus arteriosus were cut off and the ventricle was blotted dry and weighed. After one more wash in fresh buffer A it was blotted dry again and cut into smaller pieces (2 × 2 mm), which were incubated for 5 min in 2.5 ml buffer A containing 1 mg subtilisin per g ventricular muscle. At the end of incubation, 12.5 ml buffer A was added and the sample was centrifuged at 15000 g for 5 min. The resulting pellet was re-suspended in 15 ml buffer A and centrifuged at 15000 g for 5 min. The washed pellet was re-suspended in 15 ml buffer A and transferred to a glass homogenizer with a Teflon pestle. The suspension was homogenized at 500 rpm in three rounds of 1-2 seconds and centrifuged for 5 min at 1000 g. The supernatant was retained, while the pellet was re-suspended in 10 ml buffer A, homogenized at 750 rpm in three rounds of 1-2 seconds and centrifuged for 5 min at 1000 g. The two supernatants were spun at 14000 g for 10 min. Each pellet was re-suspended in 0.9 ml buffer A and centrifuged for 5 min at 2000 g. The resulting supernatants were then centrifuged for 15 min at 8000 g, and the pellets containing the mitochondria were resuspended in 0.4 ml buffer A. The isolation of mitochondria was carried out at 0-4°C, and the isolated mitochondria were kept on ice until use within 3 hours.

### Recording of ADP-stimulated oxygen consumption

Oxygen consumption of the sample was recorded in an Oroboros oxygraph-2k (Oroboros Instruments, Austria), and the rate of oxygen consumption (nmol O_2 _ml^-1 ^min^-1^) was calculated by software developed in our laboratory. To prepare permeabilized fibers for oxygraphy measurements, they were washed twice for 5 min in the R-solution used for oxygraphy. This was either the mammalian R-solution used previously [21-23] and composed for mammalian permeabilized fibers, or the new fish R-solution (see compositions below). These washes served to remove all adenine nucleotides, creatine and phosphocreatine that were present in the S-solution, in which the fibers were kept until use. For isolated cardiomyocytes and isolated mitochondria, which were kept in solutions without these components, a known volume of suspension was added to the oxygraph chamber with fish R-solution. Isolated cardiomyocytes were permeabilized in the oxygraph chamber by the addition of 20 μg ml^-1^saponin to the chamber at least 4-10 min before the first recordings. This concentration of saponin was chosen on the basis of preliminary experiments, where cells were incubated with 1 mM ADP, and the increase in respiration rate was followed in time until it reached steady state. Among the different saponin-concentrations tested, we observed that 20 μg ml^-1 ^led to a steady state respiration rate within 4-10 min (longer time at cold temperature) that was maintained for more than 30 min (results not shown).

Oxygen consumption was stimulated by stepwise increases in the ADP-concentration. For most experiments, 8 μM cytochrome c and 30 μM atractyloside was added at the end of the ADP-titration to test the intactness of the outer and inner mitochondrial membrane, respectively. At the end of the experiments, permeabilized fibers were taken out of the chamber to determine their dry weight, and allow for the expression of their respiration rate as nmol O_2 _min^-1 ^mg dw^-1^. For isolated cardiomyocytes and isolated mitochondria, an aliquot of the suspension was frozen to later determine the protein concentration by a standard bicinchoninic acid (BCA) colorimetric assay (Thermo Scientific). Knowing the volume of suspension added to the oxygraph, respiration rate was calculated as nmol O_2 _min^-1 ^mg protein^-1^.

### Solutions

S(skinning)-solution for preparation of permeabilized fibers (originally developed by [26]) was composed of (mM): CaK_2_EGTA 2.77, K_2_EGTA 7.23, MgCl_2 _6.56, imidazole 20, taurine 20, dithiotreitol 0.5, KOH 50, MES 50, Na_2_ATP 5.7, phosphocreatine 15, pH adjusted to 7.1 with KOH.

Isolation solution for isolation of cardiomyocytes [18] was composed of (mM): NaCl 100, KCl 10, KH_2_PO_4 _1.2, MgSO_4 _4, taurine 50, glucose 20, Hepes 10, pH adjusted to 6.9 with NaOH.

Buffer A for isolation of mitochondria [25] was composed of (mM): sucrose 70, mannitol 220, EDTA 1, Hepes 10, pH adjusted to 7.4 with KOH, 0.5% bovine serum albumin (BSA) added immediately before use.

Mammalian R(respiration)-solution (originally developed by [26]) was composed of (mM): CaK_2_EGTA 2.77, K_2_EGTA 7.23, MgCl_2 _1.38, imidazole 20, taurine 20, dithiotreitol 0.5, K_2_HPO_4 _3, KOH 90, NaOH 10, MES 100, glutamate 5, malate 2, pH adjusted to 7.1 with KOH, 0.2% BSA added immediately before use.

Fish R(respiration)-solution was composed of (mM): KCl 20, K-lactobionate 85, MgCl_2 _3, KH_2_PO_4 _5, EGTA 0.4, dithiotreitol 0.3, glutamate 5, malate 2, taurine 20, Hepes 20, pH adjusted to 7.1 with KOH, 0.2% BSA added immediately before use.

### Imaging of isolated cardiomyocytes

Isolated rat cardiomyocytes were provided from other experiments in our laboratory. For transmission images, the cells were mixed in isolation solution and imaged under a Nikon Ti-U microscope with a 60×/NA 1.2 water-immersed objective. For confocal images, rat and trout cardiomyocytes were kept in Ca^2+^-free Ringer and isolation solution, respectively, containing 30 mM BDM (2,3-butanedione monoxime). The sarcolemma and mitochondria were labeled by incubation for at least 15 min with the membrane potential sensitive dye Di-8-ANEPPS (5 μM; Invitrogen), and the mitochondrial potential sensitive dye Mitotracker Red (1 μM; Invitrogen), respectively. Three-dimensional confocal images (z-stacks) were acquired on a Zeiss LSM 510 confocal microscope with a 63×/NA 1.2 water-immersed objective. The images were deconvolved by software developed in our laboratory using the point spread function recorded at 543 nm [27].

### Data and statistics

Normalized kinetic parameters such as basal respiration rate in the absence of ADP (V_0_), maximal respiration rate at saturating ADP (V_max_), acceptor control ratio (ACR = V_max_/V_0_), and the ADP-affinity in terms of the Michaelis-Menten constant (K_M ADP_) were calculated using in-house software.

All values are presented as mean ± SEM, and statistical tests are described in the legends of the respective figures and tables.

## Results

### Morphology of rainbow trout compared to rat cardiomyocytes

**Figure 1:**
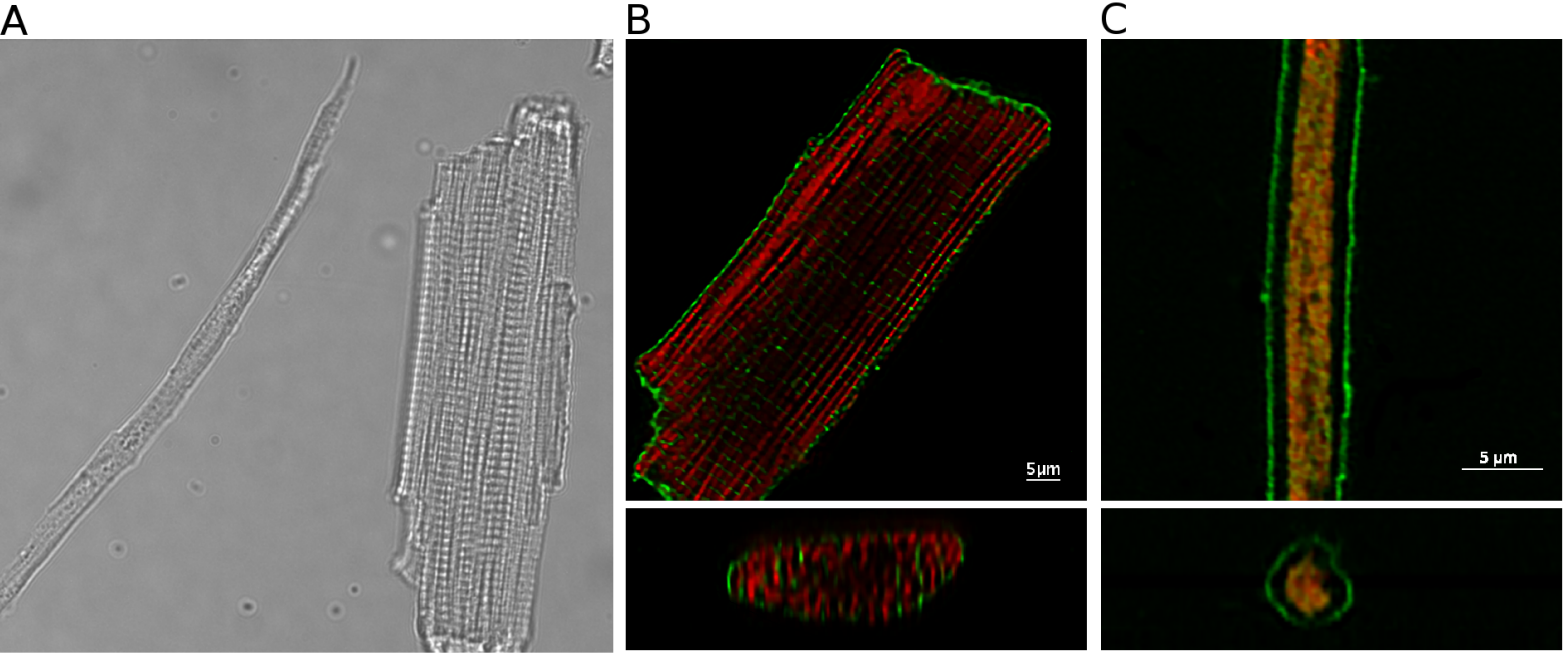
**Comparison of trout and rat cardiomyocytes**. (A) Transmission image of trout and rat cardiomyocytes next to each other in the same solution. Trout cardiomyocytes can be very long, and only half of the trout cardiomyocyte was within the camera field of view. (B, C) Deconvolved confocal images of rat cardiomyocyte (B) and trout cardiomyocyte (C) labeled with di-8-ANEPPS (green; labeling sarcolemma) and mitotracker Red CMXRos (red; labeling mitochondria). The upper image shows one image from a confocal z-stack, and lower image shows re-constructed cross section. Note different size of the scale bars.

To illustrate the size and morphology differences between trout and rat cardiomyocytes, we recorded transmission images of the two cell types together. A representative example is shown in Fig. 1A. For more detail, we recorded confocal images of cells, where the sarcolemma was labeled with the potential sensitive indicator di-8-ANEPPS (green) and mitochondria were labeled with the potential sensitive Mitotracker Red CMXRos (red). The deconvolved images and reconstructed cross sections are shown for rat and trout cardiomyocytes in Fig. 1B and 1C, respectively. These pictures illustrate that rat cardiomyocytes have several parallel rows of mitochondria that are organized as in a crystal lattice in all three dimensions [20]. This regular arrangement correlates with that of the interspersed parallel rows of myofilaments [28]. Notice also the t-tubular invaginations of the sarcolemma that extend into the center of the cell. In contrast, trout cardiomyocytes have a central core of mitochondria, which are organized rather chaotically [20]. The gap from the mitochondrial core to the sarcolemma is occupied by a single layer of myofilaments. Of note is the small diameter compared to rat cardiomyocytes and the absence of t-tubules.

### Development of a new intracellular solution for trout cardiomyocytes

**Figure 2:**
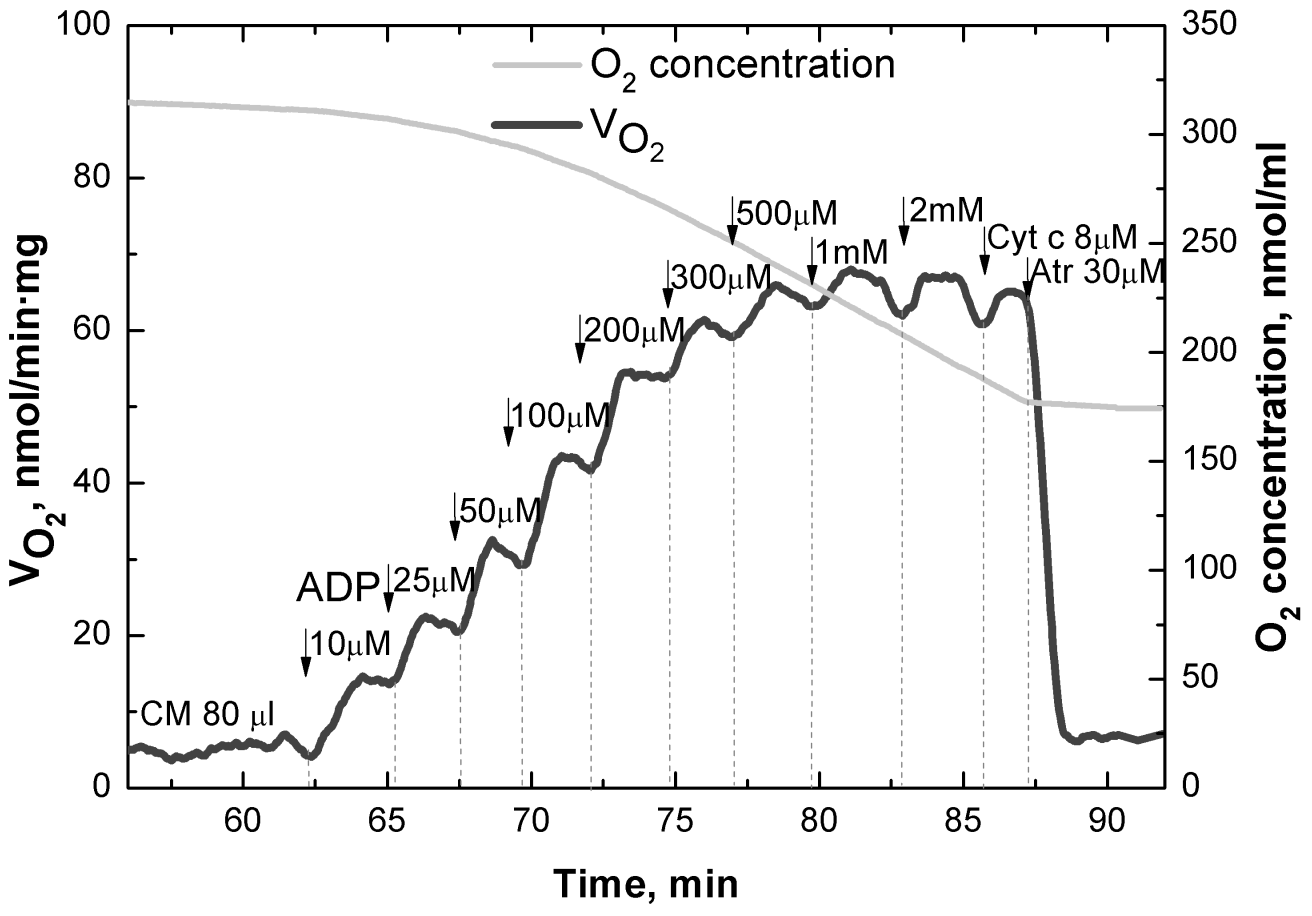
**Example of respiration of permeabilized cardiomyocytes**. Representative example recorded at 10°C in the absence of creatine showing the respiration rate of permeabilized trout cardiomyocytes (CM) during stepwise increases in ADP-concentration as indicated. Note that the respiration rate was relatively stable at each step of the ADP-titration, that cytochrome c did not increase respiration rate, and that atractyloside brought respiration rate down to the same level as the basal respiration rate before addition of ADP.

Recording of respiration of isolated cardiomyocytes was complicated with the use of "mammalian R-solution" used in previous studies on permeabilized trout cardiac fibers [21-23]. The mammalian R-solution was originally developed for studies on mammalian tissue, but permeabilized trout fibers had an acceptable ACR in this solution (Table 1). However, when isolated trout cardiomyocytes were permeabilized in this solution, their mitochondria swelled immediately followed by hypercontraction and the formation of blebs around the hypercontracted cells. We developed a new "fish R-solution" in which isolated trout cardiomyocytes maintained their morphology and showed stable steady-state respiration rates at all concentrations of ADP (Fig. 2). Interestingly, a few preliminary experiments to test this solution showed that when rat cardiomyocytes were permeabilized in the fish R-solution, they hypercontracted and oxygen consumption was virtually absent.

**Table 1:** ADP-kinetics of respiration in permeabilized fibres in mammalian R-solution

Temp	Creatine	n	V_0_	V_max_	ACR	K_M ADP_
10°C	-	5	2.28 ± 0.21	17.95 ± 2.59	7.90 ± 0.91	509 ± 57
	+	5	2.15 ± 0.25	15.88 ± 2.59	7.38 ± 0.68	252 ± 48
15°C	-	5	3.39 ± 0.45	22.17 ± 3.46	6.62 ± 0.85	399 ± 34
	+	5	3.30 ± 0.49	19.37 ± 3.69	5.82 ± 0.77	200 ± 17 *
20°C	-	5	5.48 ± 0.18	24.22 ± 1.97	4.42 ± 0.32	178 ± 32
	+	5	5.21 ± 0.45	22.71 ± 3.78	4.26 ± 0.39	129 ± 30

Respiration was stimulated by increasing concentrations of ADP, and the kinetic parameters were calculated: V_0 _(nmol O_2 _min^-1 ^mg dw^-1^) is the basal respiration rate before addition of ADP; V_max _(nmol O_2 _min^-1 ^mg dw^-1^) is the maximal respiration rate at saturating ADP; ACR = V_max_/V_0_; K_M ADP _(μM) is the ADP-concentration at which V = V_max_/2. V_max _and K_M ADP _were calculated by a hyperbolic fit of the data. Experiments were performed at three different temperatures in the absence and presence of creatine. Number of experiments is given in column n. At each temperature, results in the absence and presence of creatine were compared by a paired Students t-test. * denotes P < 0.05 significantly different in the presence of creatine. The effect of creatine at 10°C was almost significant (P = 0.0533). Temperature-dependency was assessed by a one-way ANOVA in the absence and presence of creatine, respectively. Temperature had a significant effect on V_0 _(P < 0.001), ACR (P < 0.05) and K_M ADP _in the absence of creatine (P < 0.001).

The new intracellular solution for trout cardiomyocytes was developed by trial and error. An important observation in this development was that trout cardiomyocytes maintained their overall morphology in a solution with high KCl [29]. Although their mitochondria swelled due to the high chloride concentration, they did not hypercontract. Thus, this solution was used as a starting point to approach the MIR05 solution, which is recommended for permeabilized cells [30]. The composition of the final fish R-solution is shown in Table 2, where it is compared to the mammalian R-solution, the high KCl-solution [29] and MiR05 [30]. Compared to the MIR05 solution, the main difference is that the fish R-solution contains more K-lactobionate, 20 mM KCl and no sucrose. The concentration of K-lactobionate could be varied between 20 and 130 mM without any gross effect on the oxygraph results (not shown). We chose an intermediate concentration of 85 mM, so that total K^+^-concentration was 110 mM. This is close to the concentration in the mammalian R-solution, the 108 mM in sheep purkinje cells [31], and the 118 mM in frog heart (calculated from [32] using an activity coefficient of 0.73 [33]). The presence of sucrose did not seem to affect the results, so we left it out to keep the osmolarity between that of the mammalian R-solution and MiR05 (Table 2). We speculate whether KCl was important. In some cell types, chloride transport through a mitochondrial chloride intracellular channel (mtCLIC) is believed to be important for maintaining mitochondrial membrane potential [34]. The addition of 20 mM KCl gave a total Cl^-^-concentration of 26 mM, which is very close to the 25 mM reported for chicken cultured cardiomyocytes [35] and calculated for frog ventricle (using an activity of 17.6 mM [36] and an activity coefficient of 0.7 as in [35]). With a physiological level of chloride, we could be sure that mitochondrial CK (if present) would not dissociate from the inner mitochondrial membrane, as it is known to happen in solutions with high KCl [37]. The 0.3 mM dithiothreitol in the fish R-solution ensures that the thiol-group of Cys-282 in the active site CK is reduced, which is crucial for CK function [38]. Ionic strength is intermediate between that of the mammalian R-solution and the MiR05 (Table 2).

**Table 2:** Comparison of intracellular solutions

Compound	Mammalian R-solution	KCl	MiR05	Fish R-solution
CaK_2_EGTA	2.77			
K_2_EGTA	7.23			
Imidazole	20			
K-MES	100			
KCl		125		20
Sucrose			110	
K-lactobionate			60	85
MgCl_2_	1.38	3	3	3
K_2_HPO_4_/KH_2_PO_4_	3	5	10	5
Taurine	20		20	20
Dithiothreitol	0.3	0.3		0.3
EGTA		0.4	0.5	0.4
Hepes		20	20	20
Glutamate	5	5	5	5
Malate	2	2	2	2
BSA	2 mg/ml	2 mg/ml	1 mg/ml	2 mg/ml
pH	7.1	7.1	7.1	7.1
Ionic strength	142	142	95	122
Osmolarity	288	323	330	301

Composition of the mammalian R-solution, KCl-solution (Oroboros, Austria), mitomed MiR05 (Oroboros, Austria) used for mammalian cardiomyocytes and the fish R-solution developed for the present study on permeabilized trout cardiomyocytes. All units are in mM except osmolarity, which is in mOsm.

We were able to compare the ADP-kinetics of respiration in mammalian R-solution and fish R-solution in permeabilized fibers, which survived in both solutions. The results are shown in Table 1 and 3, respectively. In agreement with previous studies, the results from permeabilized fibers had two apparent K_M ADP _values in the absence of creatine [23], but for simplicity we show only the apparent K_M ADP _obtained by fitting with a single hyperbolic equation. K_M ADP _was higher in the fish R-solution only at 15°C in the absence of creatine (P < 0.05). However, the main difference was that fibers had a better performance at high temperatures in fish R-solution: V_0 _was lower at 20°C (P < 0.001 and P < 0.01 in the absence and presence of creatine, respectively), and ACR was higher at both 15 and 20°C (P < 0.05 in the absence of creatine at both temperatures, and P < 0.01 and 0.001 in the presence of creatine at 15 and 20°C, respectively). The remainder of this article focuses on the oxygraphy data obtained using this fish R-solution.

**Table 3:** ADP-kinetics of respiration in permeabilized fibres in fish R-solution

Temp	Creatine	n	V_0_	V_max_	ACR	K_M ADP_
10°C	-	6	2.34 ± 0.21	24.85 ± 3.44	10.68 ± 1.22	783 ± 160
	+	6	2.01 ± 0.19	22.6 ± 3.83	11.03 ± 1.62	376 ± 55 *
15°C	-	5	2.78 ± 0.32	29.13 ± 3.16	10.66 ± 1.01	579 ± 64
	+	5	2.51 ± 0.08	27.25 ± 2.22	10.96 ± 1.11	301 ± 42 *
20°C	-	5	3.35 ± 0.07	28.06 ± 3.37	8.40 ± 1.05	293 ± 115
	+	5	3.37 ± 0.15	30.59 ± 1.83	9.10 ± 0.52	224 ± 42

Notation and statistical tests are as in Table 1. * denotes P < 0.05 significantly different in the presence of creatine. Temperature had a significant effect on V_0 _(P < 0.05 and P < 0.001 in the absence and presence of creatine, respectively) and K_M ADP _in the absence of creatine (P < 0.05).

### ADP-kinetics of respiration in permeabilized fibers

**Figure 3:**
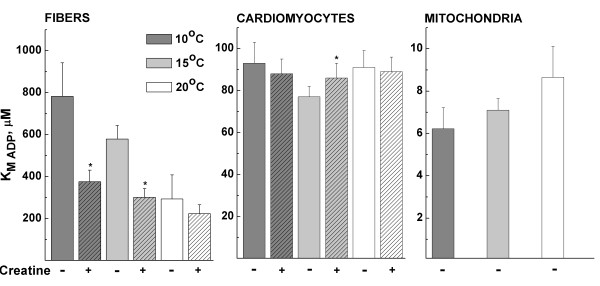
**Apparent ADP-affinity in fibers, cardiomyocytes and mitochondria**. The apparent K_M ADP _of permeabilized fibers, permeabilized cardiomyocytes and isolated mitochondria as obtained by fitting the data with a single hyperbolic equation. All experiments were done at 10, 15 and 20°C, and experiments on fibers and cardiomyocytes were performed in the absence and presence of creatine as indicated below the columns. Notice the different scales on the y-axes.

The ADP-kinetics of respiration in permeabilized fibers is shown in Table 3 and Fig. 3. V_0_, V_max _and ACR were similar in the absence and presence of creatine at each temperature. V_0 _increased with temperature, whereas V_max _and ACR were temperature-independent. K_M ADP _was lowered by creatine, and this was significant at 10 and 15°C. K_M ADP _decreased with temperature, but the temperature-effect was only significant in the absence of creatine (Table 3).

### ADP-kinetics of respiration in permeabilized cardiomyocytes

The ADP-kinetics of respiration in permeabilized isolated cardiomyocytes is shown in Table 4 and Fig. 3. V_0 _was slightly higher and ACR slightly lower in the presence than in the absence of creatine at 15 and 20°C (Table 4). However, the most important result is the apparent K_M ADP_, which is interesting to compare with that of fibers (compare in Fig. 3, note different scales on the y-axes). In contrast to permeabilized fibers, permeabilized cardiomyocytes had only one apparent K_M ADP _in the absence of creatine. This apparent K_M ADP _was lower than in fibers and similar at all temperatures. It was not lowered by creatine and even showed a slight increase at 15°C. The fact that creatine did not lower K_M ADP _was expected on the basis of a previous study [22], and suggests that trout cardiomyocytes do not have a mitochondrial CK, which is tightly coupled to respiration. The discrepancy between fibers and cardiomyocytes is discussed further below.

**Table 4:** ADP-kinetics of respiration in permeabilized cardiomyocytes

**Temp**.	Creatine	n	V_0_	V_max_	ACR	K_M ADP_
10°C	-	7	4.34 ± 0.37	52.63 ± 6.02	12.09 ± 0.67	93 ± 10
	+	7	5.40 ± 0.38 **	49.38 ± 4.70	9.11 ± 0.40 **	88 ± 7
15°C	-	8	5.79 ± 0.74	65.33 ± 5.95	12.64 ± 1.65	77 ± 5
	+	8	7.45 ± 0.55 *	63.99 ± 4.44	8.88 ± 0.78 *	86 ± 7 *
20°C	-	7	5.58 ± 1.13	63.21 ± 10.02	12.34 ± 1.08	91 ± 8
	+	7	6.04 ± 0.86	58.21 ± 9.02	9.56 ± 0.42	89 ± 7

Notation and statistical tests are as in Table 1. * and ** denote P < 0.05 and P < 0.01, respectively, significantly different in the presence of creatine.

### ADP-kinetics of respiration in isolated mitochondria

In order to make any conclusions about diffusion restrictions in trout cardiomyocytes, their apparent K_M ADP _should be compared with that of isolated mitochondria. The ADP-stimulated respiration kinetics of isolated mitochondria is shown in Table 5 and Fig. 3. In contrast to permeabilized cardiomyocytes, V_max _and ACR of isolated mitochondria increased with temperature (Table 5). In another study on rainbow trout skeletal oxidative muscle, V_max _increased but ACR decreased with assay temperature [39]. We are at present unable to explain the different temperature dependence of V_max _and ACR in permeabilized cardiomyocytes and isolated mitochondria. The main finding is that the apparent K_M ADP _of isolated mitochondria was about an order of magnitude lower than that of permeabilized cardiomyocytes (compare in Fig. 3, note different scales on the y-axes).

**Table 5:** ADP-kinetics of respiration in isolated mitochondria

**Temp**.	n	V_0_	V_max_	ACR	K_M ADP_
10°C	6	11.29 ± 2.34	46.73 ± 12.22	4.05 ± 0.28	6.22 ± 0.99
15°C	6	12.33 ± 4.89	83.18 ± 18.28	7.0 ± 0.64	7.10 ± 0.55
20°C	6	16.75 ± 2.82	131.22 ± 17.29	8.03 ± 0.51	8.65 ± 1.46

Notation and statistical tests are as in Table 1. V_max _and ACR increased with temperature (P < 0.01 and P < 0.001, respectively).

## Discussion

The main aim of this study was to verify that rainbow trout cardiomyocytes have intracellular diffusion restrictions by comparing ADP-kinetics of mitochondrial respiration in permeabilized fibers, permeabilized cardiomyocytes and isolated mitochondria. The outcomes were the following: First, isolated trout cardiomyocytes hypercontracted in solutions designed for mammalian preparations, so we had to develop a new "fish R-solution". Second, the discrepancy between fibers and cardiomyocytes suggest that previous results on trout cardiac fibers were affected by incomplete separation of the cells. Third, despite the low density of intracellular membrane structures in trout cardiomyocytes (Fig. 1), diffusion of ADP from solution to the mitochondria *in situ *is restricted. Fourth, trout cardiomyocytes seem to lack a mitochondrial CK tightly coupled to respiration. The present results on trout cardiomyocytes are very similar to those from other low-performance hearts from for example neonatal rats and rabbits [40,41].

### A new intracellular solution for permeabilized trout cardiomyocytes

It was surprising that isolated cardiomyocytes hypercontracted upon permeabilization in the "mammalian R-solution" that seemed to work for fibers. Therefore, we developed a new "fish R-solution". We were able to compare these solutions on permeabilized fibers. They had a better performance in terms of a lower V_0 _and higher ACR at high temperatures in the fish R-solution than in the mammalian R-solution (Tables 1 and 3, statistics are given in the Results section). In the fish R-solution, isolated cardiomyocytes maintained their morphology and showed stable steady state respiration rates throughout each experiment (Fig. 2). The addition of cytochrome c at the end of the experiment did not increase respiration rate showing that the outer mitochondrial membrane was kept intact. Atractyloside brought respiration rate down to the basal respiration rate recorded in the absence of ADP showing that the inner mitochondrial membrane was also intact (Fig. 2). Taken together, this validates our use of the fish R-solution for permeabilized trout heart preparations.

### Discrepancy between fibers and cardiomyocytes

Kongas et al. suggested that the high apparent K_M ADP _in fibers may be due to incomplete separation of the cells, which leads to larger diffusion distances [42]. Through the window in the oxygraph chamber, we observed that fibers tend to cluster on top of the magnetic stirrer during oxygraphy measurements. This is avoided with permeabilized cardiomyocytes, where all cells are completely separated, and we observed them to be evenly distributed in the oxygraph solution. Incomplete separation of the cells is not a problem in permeabilized fibers from rat heart, which have the same apparent K_M ADP _as permeabilized cardiomyocytes [6,43]. However, studies on trout fibers gave paradoxical results inasmuch as creatine decreased the apparent K_M ADP _[21,23], although there seemed to be no mitochondrial CK tightly coupled to respiration [22].

The present experiments showed that K_M ADP _in trout fibers is higher than in cardiomyocytes. It depends on temperature and is lowered by creatine, but this is not the case for permeabilized cardiomyocytes. This discrepancy suggests that results from trout fibers were affected by incomplete separation of the cells. It is difficult to separate the cells more in trout heart permeabilized fibers, because the tissue is very soft and fragile compared to rat cardiac tissue. We observed that a more thorough dissection led to mechanically damaged fibers with unstable respiration rates and a low ACR.

It has been questioned whether the high apparent K_M ADP _in fibers could be caused by unstirred water layers immediately adjacent to the surface of the fibers or cells [42]. From the same line of thought follows whether fibers have a higher K_M ADP _than cardiomyocytes, because they tend to cluster and thus have thicker unstirred layers than cardiomyocytes. However, two observations from our data strongly suggest that unstirred layers are not the main cause of diffusion restriction. First, the apparent K_M ADP _should increase with temperature, because metabolic rate (Q_10_~2 [44]) would be more limited by diffusion speed (Q_10_~1.3 [45]) at higher temperatures. In contrast, we observe a decrease in K_M ADP _with temperature in fibers (Fig. 3A) and no effect of temperature in cardiomyocytes (Fig. 3B). Second, stimulation of cytosolic CK with creatine would also have an effect in isolated cardiomyocytes. In contrast, creatine only lowers K_M ADP _in fibers (Fig. 3A) and not in cardiomyocytes (Fig. 3B). Thus, neither the higher K_M ADP _in cardiomyocytes compared to isolated mitochondria nor the higher K_M ADP _in fibers compared to cardiomyocytes can be explained by unstirred layers.

One possible explanation for the higher and temperature-dependent K_M ADP _in fibers could be that perforated sarcolemma is still left to restrict ADP-diffusion between cells. This may become stiffer at colder temperatures and restrict ADP-diffusion more resulting in a higher K_M ADP_. This would also explain why creatine lowers apparent K_M ADP _in fibers but not cardiomyocytes. Diffusion across the barriers formed by the sarcolemma in fibers is facilitated by stimulation of cytosolic CK, whereas this is not the case in permabilized cardiomyocytes. This hypothesis could be confirmed by experiments using raster image correlation spectroscopy (RICS) to study diffusion restriction. We recently used this method to determine the diffusion of fluorescent ATP in adult rat cardiomyocytes. Diffusion was anisotropic, being 2 times slower in the longitudinal direction and 3.5 times slower in the transverse direction compared to solution [46]. However, this method only gave information on the overall diffusion coefficients in cells versus solution. It needs further development before it can be used to distinguish diffusion coefficients in different cellular compartments and localize the additional diffusion barriers in fibers compared to cardiomyocytes. Although the exact cause of the discrepancy between fibers and cardiomyocytes is not completely resolved, we conclude that trout cardiac fibers, which have been used in previous studies [21-23], do not give reliable information about intracellular diffusion restrictions in trout cardiomyocytes.

### ADP diffusion restrictions and role of creatine kinase in rainbow trout cardiomyocytes

The apparent K_M ADP _of permeabilized trout cardiomyocytes is independent of temperature and not lowered by creatine. The K_M ADP _is ~80-90 μM, and this is about ten times higher than of isolated mitochondria (Tables 4 and 5 and Fig. 3). Thus, diffusion of ADP from the medium to the ANT in the inner mitochondrial membrane is restricted. The present study does not give any precise information as to the cause and localization of diffusion restrictions. The magnitude of diffusion restriction in terms of K_M ADP _is about three times smaller in trout than in rat cardiomyocytes. Thus, t-tubules and SR may still restrict diffusion in rat cardiomyocytes. From the present experiments, we can only conclude that they are not the only cause of diffusion restriction.

Our results suggest that trout heart lacks a mitochondrial CK, because creatine does not lower the apparent K_M ADP _(Table 4 and Fig. 3). This is in agreement with a previous study [22]. Additionally, the positive charge of mitochondrial CK in rat heart seems to be a prerequisite for binding to the inner mitochondrial membrane [9], but preliminary experiments with isoelectric focusing suggest that trout heart does not express a positively charged CK isoform (R. Birkedal, unpublished observation). Further experiments will be required to determine whether trout heart expresses a mitochondrial CK. However, the expression of mitochondrial CK does not always correlate with functional coupling to respiration [47]. In tissues such as rat ventricle and oxidative skeletal muscle, where mitochondrial CK (Mi-CK) is tightly coupled to respiration [48], diffusion restriction by the outer mitochondrial membrane will enhance this coupling [49]. Indeed, mathematical modeling of data from rat heart suggests a moderate restriction of diffusion by the outer mitochondrial membrane and a stronger restriction of diffusion in the cytosol, probably formed by SR together with crowding of cytoplasmic proteins [50]. However, diffusion restriction by the outer mitochondrial membrane seems unfavorable in tissues that lack a tight coupling of mitochondrial CK to respiration. This is the case for adult rat atrium, which expresses Mi-CK [47,48], neonatal rat and rabbit ventricle, which do not express Mi-CK [40], and according to the present results also trout ventricle. More experiments and development of a mathematical model for trout cardiomyocytes are needed to quantify diffusion restriction by the outer mitochondrial membrane and cytosolic factors, but the present results argue against diffusion restriction by the outer mitochondrial membrane in trout cardiomyocytes.

### Rainbow trout as a model animal to study diffusion restrictions in low-performance hearts

The magnitude of diffusion restriction and the importance of CK in cardiomyocytes seem to relate to metabolism and cardiac mechanical performance. Recent studies have addressed diffusion restriction and metabolic regulation in beating and non-beating HL-1 cardiomyocytes in culture derived from AT-1 mouse atrial tumor cells [51]. Both express cardiac isoforms of connexin, desmin and several ion channels [52,53], but their morphology is vastly different. The cells are flattened out against the two-dimensional surface on which they are grown. Mitochondria form a reticular network, which seems to have some relation to the sarcomeres found in beating cells [52,54]. In non-beating cells, sarcomeres are absent [53]. Their metabolic phenotype is glycolytic [55], and the apparent K_M ADP _is low (~50 and 25 μM for beating and non-beating cells, respectively) [54]. Indeed, the characteristics of cultured cardiomyocytes are affected by contractile activity and mechanical load [56], and it seems that for studies of intracellular diffusion restrictions, they cannot yet replace cardiomyocytes that are freshly isolated from working hearts.

Rainbow trout cardiomyocytes seem to have a phenotype that is intermediate between cultured cardiomyocytes and adult mammalian ventricular myocytes. Interestingly, they have these characteristics in common with cardiomyocytes from other low-performance hearts, e.g. neonatal rats and rabbits. Their apparent K_M ADP _is close to 100 μM and they lack Mi-CK coupled to respiration [40,41]. Compared to adult mammalian cardiomyocytes, they show greater hypoxia tolerance [57] and rely more on glycolytic energy production [40,58], but less so than the cultured cells. In addition, trout and neonatal rabbit cardiomyocytes have similar morphology (compare Fig. 1 with [59,60]) and possibly also excitation-contraction coupling [61,62]. Thus, it seems that cardiomyocytes from low-performance hearts are very similar independent of species. It is likely that knowledge about the role of diffusion restrictions and CK in trout cardiomyocytes can be extrapolated to other low-performance hearts.

## Conclusions

A new solution was developed for permeabilized trout cardiomyocytes. The results suggest that previous data from permeabilized trout heart fibers were affected by incomplete separation of the cells. This seems to be specific for trout cardiac fibers. The higher apparent K_M ADP _in fibers could be due to remains of sarcolemma between cells in a fiber bundle. However, even in permeabilized trout cardiomyocytes, which have a very small diameter and low density of intracellular membrane structures, diffusion of ADP from the surrounding medium to the ANT in the inner mitochondrial membrane is restricted. Our results exclude the hypothesis that the main cause of this restriction is unstirred layers. Trout cardiomyocytes do not have a mitochondrial CK coupled to respiration. This argues against diffusion restriction by the outer mitochondrial membrane. Whereas it may be important in rat cardiomyocytes, it is more likely that the diffusion restrictions in trout cardiomyocytes reside in the cytosol. The characteristics of rainbow trout heart are very similar to those of other low-performance hearts such as neonatal rat and rabbit hearts. Most probably, rainbow trout can be used as a model animal to study further the localization and physiological importance of intracellular diffusion restrictions in low-performance hearts in general.

## List of abbreviations

ACR: acceptor control ratio (= V_max_/V_0_); ADP: adenosine diphosphate; ANT: adenine nucleotide translocase; ATP: adenosine triphosphate; BSA: bovine serum albumin; ICEU: intracellular energetic unit; K_M ADP_: mitochondrial Michaelis Menten constant for ADP; mtCLIC: mitochondrial chloride intracellular channel; SR: sarcoplasmic reticulum; SERCA: sarco-endoplasmic reticulum Ca^2+^-ATPase; V_0_: basal respiration rate before addition of ADP; V_max_: maximal respiration rate.

## Authors' contributions

NS performed all the experimental work. MV participated in design of the study and revision of the manuscript. RB conceived, designed and coordinated the study, and drafted the manuscript. All authors have read and approved the manuscript.
